# Milestones

**Published:** 1920-01-24

**Authors:** 


### MILESTONES.

HAVE you ever, reader, in the golden age, journeyed along one of the great highways, counting the milestones? It is a childish occupation, and children love counting. Directly one is passed it is forgotten. It was number this or that, and the thing to do is to wait for the next. In the interval there are a variety of distractions—birds and flowers, toilers in the fields—and so on to the journey's end. It is only when we put away childish things that we begin to realise that these milestones are limited in their number, and that the stones themselves are of no importance, but that the intervals mean everything.

We have just passed a milestone. Please note. The two nineteens have gone the way of all flesh. They mark the end of a frightful human cataclysm such as has not been experienced since Noah and his caravanserai planted their feet on a storm-swept world. We do not yet see things as they are. Men and women are like trees walking. The one fact that is obvious is that there is an enormous amount of work to be done. The prophets tell us that we are witnessing the dawn of a new world, but people who are not prophets realise that the ruins of the great conflagration must be cleared away before the new world can be constructed, and the inward monitor prompts that the milestones are passing.

We have just passed one!

It is not the first time that I have been told that I lack patience. I do not know whether it is the echo of a previous incarnation, but it seems to me that, looking backward, there has always been a little devil at my elbow whispering "patience." And I have always impatiently asked, "Why?" But I have never received an answer, unless it is an answer to repeat "Be patient!"

To be frank, I do not think that any of us has any time for patience. We are all swinging along, old men and babes, kings and beggars, willy-nilly, to our long home. The reason why I am not patient is very strong. I cannot afford it. I am one of the *dramatis personæ* of this act, and so are you. The next fall of the curtain may mark our final disappearance from the stage. If there is anything that you and I can accomplish—ever—it seems to me that there is only one word, and that word is "Now."

What is there to wait for?

This question is not shot at random. It is put in dead seriousness to the advocates of patience, and claims an answer. I can well understand that if a man is dying, people who are interested ought to postpone calling in the undertaker too soon. It has occasionally happened that dying men outlived some of their healthy friends. I am concerned with nursing reform, and most people appear to be agreed that such is necessary. In this connection I ask, What is there to wait for? Is anything likely to occur in 1921 or 1922 or 1950 that will justify nursing reform more than in 1920? Nursing reform, thank God! does not necessarily depend on an Act of Parliament. If it did, there would be a valid reason for indefinite postponement, and all the little devils might join in a war dance to my undoing. Acts of Parliament are certainly cases for patience.

I want to bring the question home, Is the emancipation of the nurse to be postponed until you and I and the nurse are all dead and buried? There is Lord Knutsford. He says it is "impossible" that the nurse shall have a forty-eight-hours' week. Had he said it is difficult, extremely difficult, I should have agreed. But I can look forward with absolute confidence to a time when the nurse shall enjoy a forty-eight hours' week. I have no more doubt of it then I have of my own existence. I may rest in a forgotten grave, and the patients in the "London" Hospital may contemplate a statue of Lord Knutsford and wonder who he was and what he did. Some matron of the future may refer to him as the Governor who regarded a forty-eight hour week as impossible. [And, if she is a wise woman, she may add that few, if any, of his contemporaries thought likewise.]

Of all others, this doctrine of patience is to me the most dangerous. It is, in essence, selfish, and signifies—"do not worry me now—I cannot be bothered; put it off to a more convenient season."

What is wrong with NOW? Of course if it is not now, it becomes a matter for patience, a matter, in other words, for an Act of Parliament, and the abolition of the hospital voluntary system, of which this generation is so proud. Modern days have seen the end of many voluntary systems. The voice of the toiler was for many generations a voice in the wilderness. Now, if you please, or if you please not, you must consider him. In some cases the toiler is losing his head. Having advanced so far, he is trying to assume the mastery. In the long procession of toilers the nurse comes last. By common consent hers is the hardest work, and in importance second to none. She is conserving and preserving the nation's health.

Is this right? Is it reasonable? Is it tolerable?

The problem of the nurse can never be solved until it is put to the British people frankly and without reserve. I am optimist enough to believe that this will be done very soon, and that the pioneers will include in the foremost ranks the very men who now regard the solution as impossible.

What a fine programme for 1920! IERNE.

